# Short-Term Effectiveness and Reduction in Insulin Requirements in Patients With Type 2 Diabetes Treated With IdegLira in a Real-World Setting

**DOI:** 10.3389/fendo.2022.828607

**Published:** 2022-04-28

**Authors:** Alex Ramírez-Rincón, Carlos E. Builes-Montaño, Jaime A. Hincapié-García, Victor M. Blanco, José F. Botero-Arango

**Affiliations:** ^1^ School of Medicine, Universidad Pontificia Bolivariana, Medellín, Colombia; ^2^ Endocrinology Department, Clínica Integral de Diabetes, Medellín, Colombia; ^3^ School of Medicine, Universidad de Antioquia, Medellín, Colombia; ^4^ Endocrinology Department, Hospital Pablo Tobón Uribe, Medellín, Colombia; ^5^ Clinical Pharmacology, Pharmaceutical Promotion and Prevention Group, Faculty of Pharmaceutical and Food Sciences, Universidad de Antioquia, Medellín, Colombia; ^6^ School Medicine, Pontificia Universidad Bolivariana, Medellín, Colombia

**Keywords:** type 2 diabetes, insulin degludec, liraglutide, IDegLira, real-world evidence (RWE)

## Abstract

**Background:**

Type 2 diabetes mellitus (T2DM) is a chronic, highly prevalent disease with a significant impact on health. Appropriate treatment requires effective and timely escalation to achieve metabolic control. To evaluate the effectiveness and safety of IDegLira on adults with T2DM previously treated with oral antidiabetics and/or insulin in a real-life setting.

**Methods:**

An observational study in a real-world setting was conducted. Patients were selected from the outpatient clinic of two centers dedicated to specialized diabetes care. Main outcomes were HbA1c, body weight, insulin dose changes, hypoglycemia, and other adverse events.

**Results:**

67 T2DM patients treated with IDegLira were monitored between 3 and 7 months. At the end of foll ow-up, the median change in HbA1c was -1.05% (CI95% -1.45, -0.65), and a decrease in insulin requirement was also observed (mean difference -10 TDD units (CI95% - 17 to -2.5). No treatment discontinuation was reported, hypoglycemia events were reported in 3 patients at the end of follow-up versus 8 patients at baseline.

**Conclusions:**

This real-life study shows the effectiveness in glycemic control of IDegLira use in T2DM patients who do not achieve goals with other therapies, with an adequate safety profile. The findings need to be confirmed with evaluation of therapeutic results in larger cohorts.

## Introduction

Type 2 diabetes mellitus (T2DM) is a chronic disease with a great impact given its increasing prevalence, which is close to 8.5% worldwide, and the associated premature mortality (1, 2). Therapeutic efforts in T2DM have focused on a series of organized, individualized steps with the purpose of controlling the elevation of basal and postprandial blood glucose, resulting from the deregulation of the metabolism of carbohydrates, lipids and proteins. Evidence has shown that appropriate and timely therapeutic intensification reduces the risk of micro- and macrovascular complications in T2DM (1). However, many patients remain in suboptimal or non-staggered treatments, especially with insulin, out of fear of hypoglycemia and weight gain (2).

Injectable medications to treat diabetes include GLP1 receptor agonists (arGLP1), incretin mimetics that decrease basal and postprandial blood glucose (3). These drugs stimulate the production of endogenous insulin and decrease the secretion of glucagon, thus acting in seven of the eleven metabolic alterations documented in diabetes (4). It has also been shown that the arGLP1, in addition to the hypoglycemic effect that leads to average reductions of glycated hemoglobin (HbA1c) between 0.9 and 1.4%, generate weight reduction due to actions in the suppression of appetite (3–5).

IDegLira is the combination of a second-generation long-acting insulin analogue (Degludec) with an arGLP1 (Liraglutide) (6), whose objective is to enhance the effectiveness of insulin as a result of complementary mechanisms of action (7), with the benefits of arGLP1 with a decrease in HbA1c and weight. The safety and efficacy of this drug in T2DM patients were evaluated in the DUAL program, in DUAL V study it was compared with basal insulin alone, finding a significant decrease in HbA1c and additionally weight loss and lower risk of hypoglycemia (8). The DUAL study VII compared the use of basal-bolus insulin versus IDegLira, showing the non-inferiority of the latter in declining HbA1c, with a lower risk of hypoglycemia and significant weight loss in contrast to weight gain in the basal-bolus group. Additionally, a significantly lower insulin requirement (TDD - Total Daily Dose) in the intervention arm (40 IU versus 84 IU, mean difference -44.5, CI95% -48.3, -40.7) (9).

Although clinical trials are the ideal scenario for demonstrating the efficacy and safety of any drug, strict selection criteria and the controlled environment limit the generalization of results and the possibility of exploring some important outcomes such as formulation patterns and their interaction with health systems. Additionally, those who evaluate insulin usually follow the *treat to target* strategy and some outcomes, such as reduction in insulin dose, which are difficult to estimate. This study aimed at evaluating the effect of intensification with IDegLira on HbA1c control, change in body weight, and variation in insulin dose in T2DM patients in a real-life scenario.

## Methods

An observational, real-world study was conducted across two sites in Colombia: Clínica Integral de Diabetes (CLDI), a center specialized in diabetes within a structured and comprehensive model of chronic care, and Hospital Pablo Tobón Uribe, a reference center, both in the city of Medellin-Colombia. We included all adult patients with T2DM treated with insulin degludec/liraglutide in 2019 and 2020 and with at least three-months follow-up. Patients were selected by identifying the insulin degludec/liraglutide prescription. Pregnant women and patients with liver function insufficiency or chronic kidney disease with glomerular filtration of less than 30 ml/min were excluded. As it is an observational study, titration was performed according to treating physician decision.

The primary outcome was the change in glycated haemoglobin (HbA1c) level. Secondary outcomes included changes in body weight and insulin dose, self-reported hypoglycemia events and other adverse events reported by patients with the use of IDegLira.

The information was extracted from electronic medical records and stored in a database with double validation as quality control. Demographic data, including age, sex and educational level; clinical variables related to the duration of diabetes, weight (in kilograms), body mass index (BMI), glycated haemoglobin (HbA1c) level, basal glycemia, comorbidities, antihyperglycaemic regimen (doses and type of insulin, other therapies for diabetes), follow-up time, and hypoglycemic events were recorded. Clinical variables of interest were measured at therapy baseline and until the end of follow-up period.

The study was conducted based on the principles of the Declaration of Helsinki and it was approved by our institutional ethics committee.

Continues variables are presented as medians and interquartile range, qualitative ones as absolute and relative frequencies. The median of the difference for quantitative measures was estimated using the Wilcoxon rank sum test and nonparametric confidence interval were computed. The results on the change of HbA1c were stratified by previous treatment. To assess the impact of missing information on the outcomes a sensitive analysis was planned through a multiple imputation technic by bootstrapping. Data was analyzed using R statistical software.

## Results

This study included 67 patients who met the selection criteria. Baseline demographic and clinical characteristics of patients are described in **Table 1**. In the patient cohort, men were slightly predominant (52.2%) and the age median was 64 years (SD 15.8). Most of the patients had comorbilities such as dyslipidemia and/or hypertension; half of the patients had been diagnosed with T2DM for over 13 years and used oral antidiabetics (OAD), with a predominance of iSGLT2 and GLP1-RA. Prior to treatment with IDegLira, 51 patients received insulin, on average 30 U (IQR 18) of basal insulin and 23 U (IQR 18) of prandial insulin. The patients had a median body mass index (BMI) of 28.4 (range 18.4 to 43.5). The median of the initial HbA1c was 8.5% (range 6.5% to 13.2%).

**Table 1:** Baseline characteristics of patients included in this study.

	Overall (N=67)
**Age (y), median [IQR]**	64.0 [15.8]
**Sex, male, n (%)**	35 (52.2%)
**Education level**	
**Elementary or intermediate**	17 (25.4%)
**Undergraduate**	17 (25.4%)
**High school**	15 (22.4%)
**Vocational education**	14 (20.9%)
**Postgraduate**	3 (4.5%)
**High blood pressure, n (%)**	35 (52.2%)
**Dyslipidemia n (%)**	45 (67.2%)
**Diabetes duration (years), median [IQR]**	13.0 [11.0]
**Oral antidiabetics, n (%)**	64 (95.5%)
**Metformin**	44 (65.7%)
**SLGT2 inhibitor**	24 (35.8%)
**GLP1-RA**	23 (34.3%)
**DPP4 inhibitors**	18 (26.9%)
**Sulfonylureas**	2 (3.0%)
**HbA1 < 8%, (% patients)**	27%
**HbA1 < 7.5%, (% patients)**	10%
**HbA1 < 7%, (% patients)**	4%

BMI, Body mass index; CI, confidence interval; IQR, Interquartile range; N, number of patients in each strata. IQR, Interquartile range; DPP4, Dipeptidyl Peptidase-4; GLP1-RA, Glucagon-like peptide-1agonists; SGLT2, sodium-glucose cotransporter type 2.

**Figure 1:**
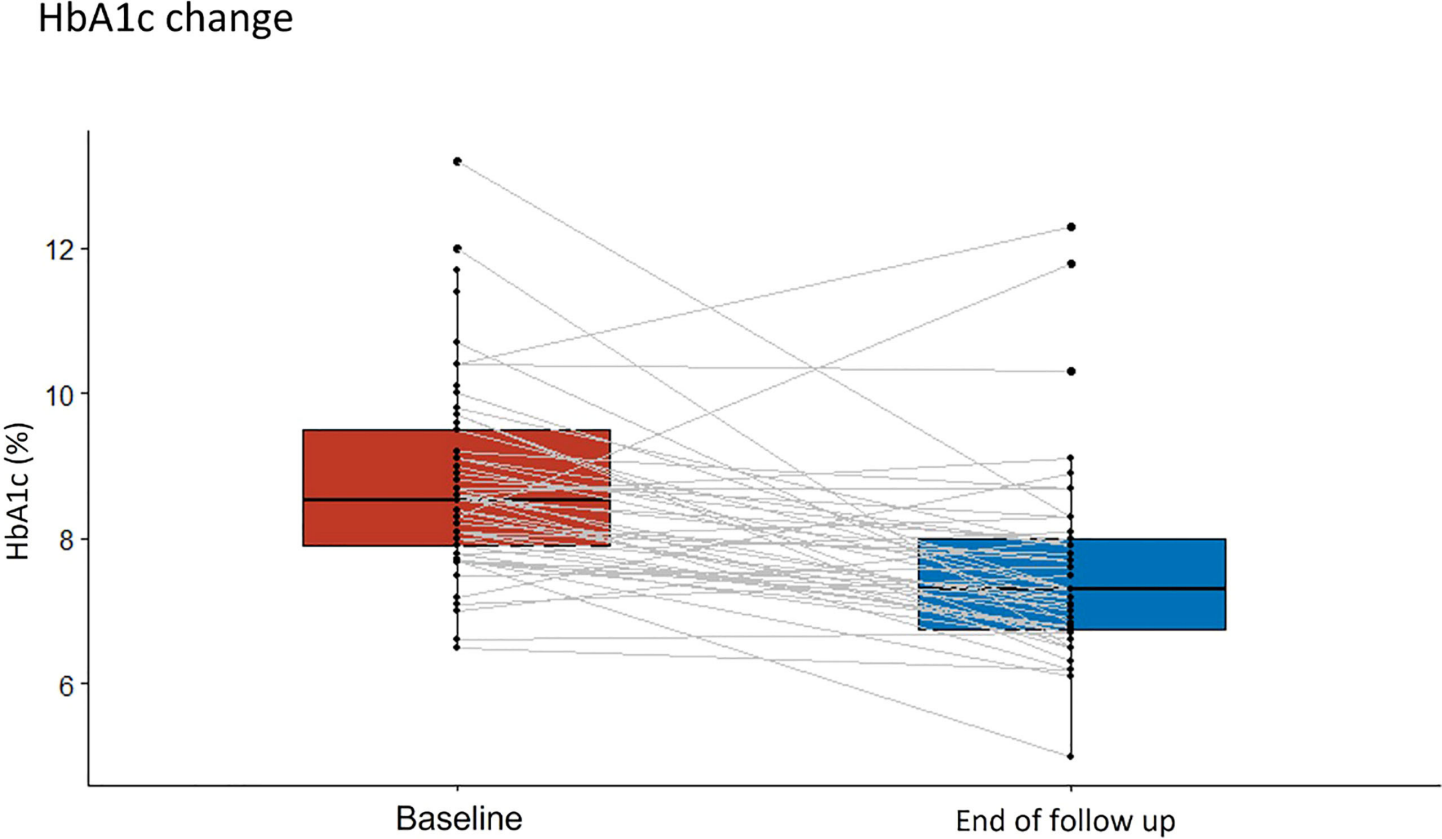
Change in median glycosylated hemoglobin (HbA1c) from the beginning of the observation period to the therapy follow-up with insulin degludec/liraglutide (IDegLira).

**Figure 2:**
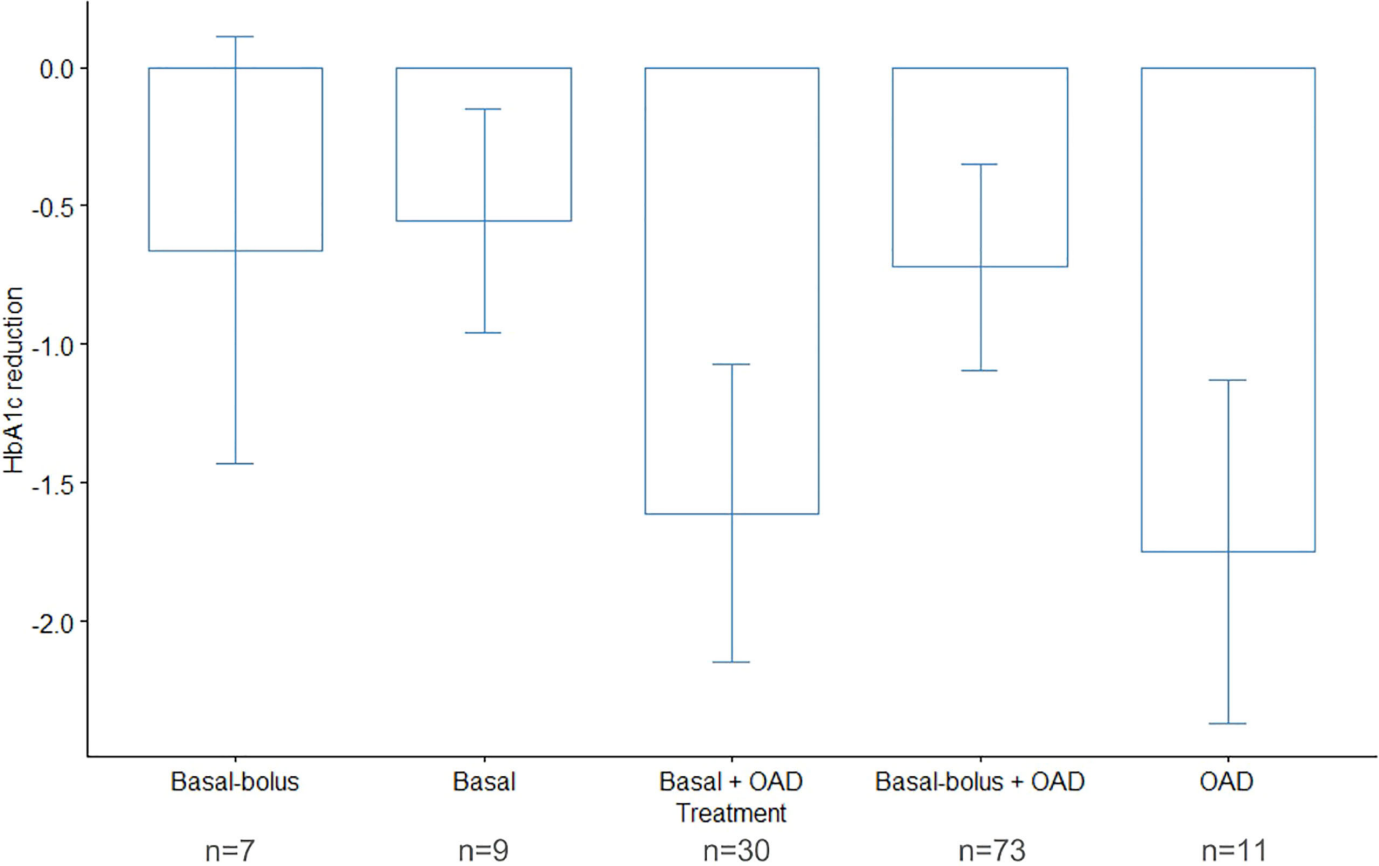
Change in median glycosylated hemoglobin (HbA1c) during follow-up, by hypoglycemic therapy prior to insulin degludec/liraglutide (IDegLira). HbA1c, glycated hemoglobin; N, number of patients in each strata; OADs, oral antidiabetic drugs.

**Figure 3:**
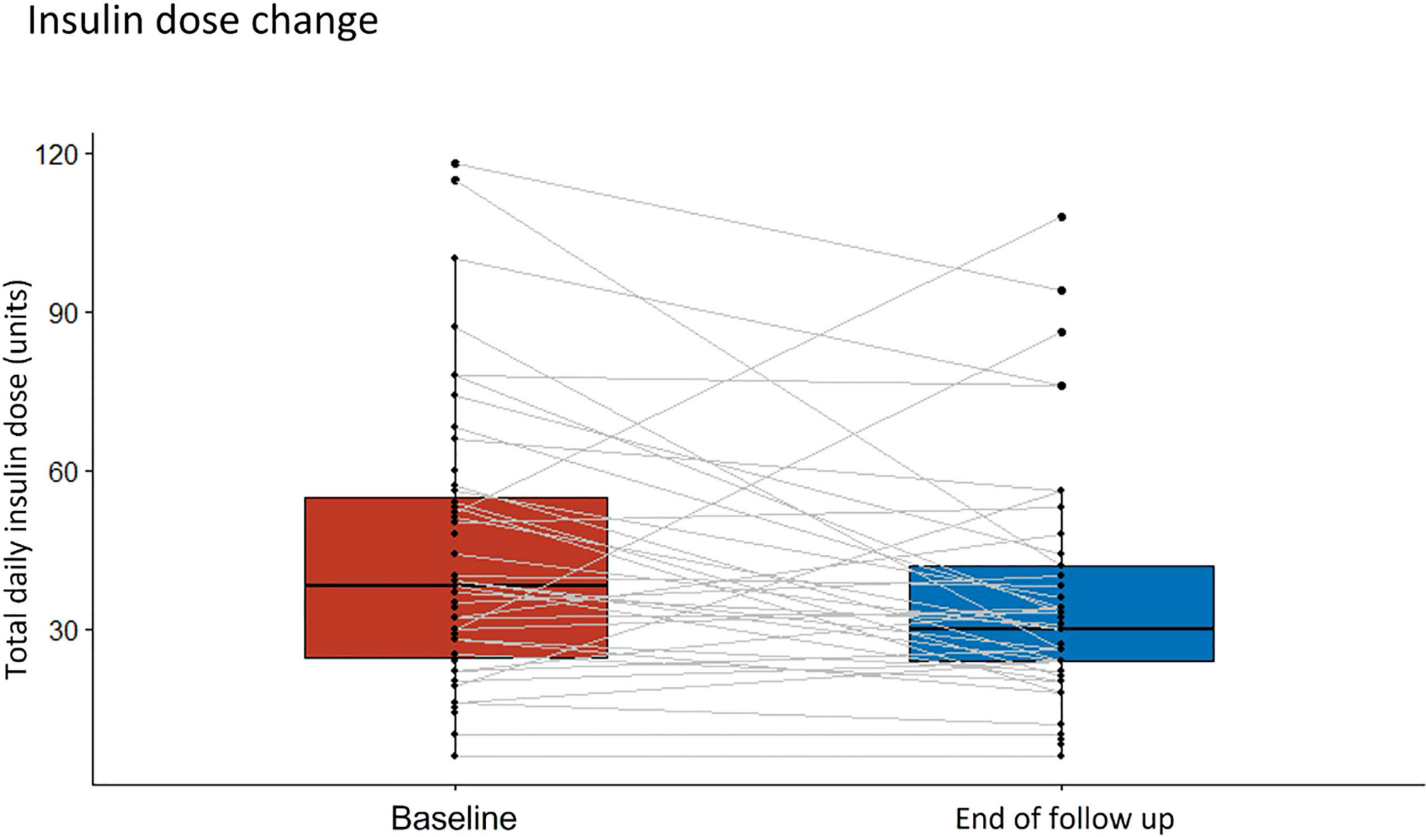
Change in median daily insulin dose from the beginning of the observation period to the therapy follow-up with insulin degludec/liraglutide (IDegLira). *TDID, total daily insulin dose*.

Patient follow-up time ranged from 3 to 7 months. No patient needed titration to the maximum dose of the medication, and the highest dose required was 40 units in 5% of patients. Baseline and follow-up data for outcome of interest measures are shown in **Table 2**. At the end of the follow-up period, there was a decrease in levels of HbA1c, with 55% of patients reaching levels of 8% or less versus 27% of patients with these levels at baseline. The statistically significant reduction of the median of HbA1c was about 1.05% (CI95% -1.45, 0.65) (see **Figure 1**), and the largest reductions (median greater than -1.5%) were seen in patients who were treated with OAD with or without basal insulin (see **Figure 2**). Insulin requirement decreased at follow-up by a median of 10 TDD units (see **Figure 3**); the reduction was higher in the dose of prandial insulin (-5 U; CI95% -16, 17) than at basal insulin dose (2 U; CI95% -1.5, 6). At the start of treatment, 43% of the patients were under basal bolus regime, but only 24% of them at the end of follow-up. Treatment with IDegLira was not associated with any significant change in weight.

**Table 2:** Change in clinical and biochemical parameters after treatment with insulin degludec/liraglutide.

Characteristic	At baseline	At end of follow up	Median difference (CI 95%)	p value
**HbA1c (%), [IQR]**	8.5 [1.6]	7.3 [1.24]	- 1.05 (- 1.45 to - 0.65)	<0.0001
**Body weight, Kg, [IQR]**	75 [18.4]	73 [15]	0.5 (- 0.25 to 1.3)	0.13
**BMI (Kg/m²), [IQR]**	28.4 [5.45]	28.4 [6]	0.5 (0.18 to 1.0)	0.0024
**Total daily insulin dose (units), [IQR]**	38 [30.5]	30 [17.5]	- 10 (- 17 to -2.5)	0.01
**HbA1 < 8%, (% patients)**	27%	55%	NA	
**HbA1 < 7.5%, (% patients)**	10%	41%	NA	
**HbA1 < 7%, (% patients)**	4%	27%	NA	

BMI, Body mass index; CI, confidence interval; IQR, Interquartile range; N, number of patients in each strata.NA, not applicable.

Regarding adverse events, at baseline, 8 patients reported episodes of hypoglycemia versus 3 patients at the end of follow-up with therapeutic adjustment, in addition to no reports of severe hypoglycemia. No treatment discontinuation was reported during the observation period. At follow-up, all patients continued using the prescribed medication at a dose of 16 U (IQR 4).

In the sensitivity analysis, no difference in direction or magnitude was found in the results of change in HbA1c, body weight or insulin dose.

## Discussion

Baseline characteristics of the population evaluated in this study are like real-life experiences recorded in other studies. Most reports describe a range between 56 and 64 years (10–12), in this case the median age of 64 years as it is reported by Zoltan et al. in 2019 (10). The time of evolution of diabetes over a decade in most patients is similar to other published studies with values between 11 (10) and 13 years of disease progression (11). With regard to glycemic control, in this group of patients, the baseline mean of HbA1c was 8.53%, other real-life studies report ranges between 6.42% and 9.9% (10, 13). The variability of the baseline levels in other studies is explained according to the research objective, such as evaluating the effectiveness of a drug in real-life experience or simplifying the management of complex insulin schemes in which glycemic control was not a selection criterion for participants.

Reduction in insulin TDD is a difficult outcome to evaluate in a clinical trial, however, it has been explored in real-life scenarios, as in this study, documenting an important reduction. In the DUAL randomized clinical trial program, Ideg/Lira compared to basal/bolus (BB) time-intensification produce a reduction in TDD of 43.7 IU (BB 84.1 IU/day vs insulin degludec/liraglutide 40.4 IU/day) (9). In the DUAL program patients had a poor metabolic control under a basal insulin regime, in clinical practice starting Ideg/Lira in better controlled patients may not generate high expectations. When reviewing this outcome in similar real-life studies the findings are heterogeneous at best with reductions from 0.23 IU/day in relatively well controlled population being reported (10) and greater differences such as 5.5 IU/day (12) but with baseline levels greater than HbA1c (mean 9.8%).

In a therapeutic scenario as complex as the pharmacological treatment in T2DM, multiple aspects should be considered in decision-making, particularly important in highly demanding treatment schemes such as basal/bolus and basal/plus. In this context, the results of this study with a reduction in TDD in a real-life scenario that achieves adequate metabolic control with IDegLira, without significant weight gain and in the absence of severe hypoglycemia, are of great importance.

The proven effectiveness in clinical trials of the IDegLira development program (8, 9, 14–19) has also been reported in real-life scenarios with reductions in HbA1c in the range of 0.3% to 2.2% (20) [preserving the tendency of the Bloomgarden effect (21)]: the higher the basal level the greater the control effect on HbA1c. The finding of a reduction close to one percentage point in HbA1c in people whose initial median was 8.5% is interesting given the time of disease progression in this group of patients as well as the high proportion of those treated with insulin and the low dose of insulin degludec/liraglutide reported at the end of follow-up. The high proportion of patients who achieved metabolic control with a reduction of HbA1c to values below < 7%, 7.5% and 8%, of high clinical value in a real-life scenario is highlighted.

The effect on weight in the patients included in the study, in whom there was no significant reduction in BMI, was also an important finding, in possible relation to the characteristics and baseline therapeutic regimen of the subjects included. Real-life studies have reported mean weight reduction in the range of 0.7 to 6.76 kg (10, 12) with the intervention in question. Although hypoglycemia outcomes were not predetermined for analysis, no patients reported severe hypoglycemia during the study period. This finding is expected, and the lower incidence of hypoglycemia is confirmed with the use of a new generation long-acting insulin analog (insulin degludec/liraglutide) co-formulation versus other therapeutic regimens such as basal/plus or basal/bolus. Other real-life work has reported a decrease of up to 82% in the appearance of hypoglycemia in the population involved (11).

Several factors have been documented as fundamental in the therapeutic adherence of T2DM patients, and this remains a current challenge against the low rates of optimal glycemic control in this population. This study showed no discontinuation of therapy during follow-up time, consistent with documented persistence with IDegLira in diabetic adults previously treated with other hypoglycemic regimens, with better adherence and glycemic results from those using OAD and switched to the fixed-dose combination (22). This is important both for glycemic control and for reducing the risk of all-cause mortality and hospitalization in T2DM patients, according to evidence from observational studies (23), and the demonstrated reduction in health care costs following adequate therapeutic adherence in these patients (24).

This study has some limitations inherent to its observational nature. This study is neither controlled nor blinded and is based on the experience of two health care centers. The potential effects of other factors on glycemic outcomes, such as adjustments of other hypoglycemic medications, are not further estimated. The sample size is small and follow-up time was variable which also potentially introduces other bias over the outcomes evaluated. The results of this study suggest the effectiveness of the use of IDegLira in T2DM patients who have not made progress with other therapies in practice, with better glycemic control and reduced insulin requirements. The findings should be confirmed in cohorts with a larger number of patients and broader evaluation of therapeutic outcomes and safety.

## Data Availability Statement

The datasets used and/or analysed during the current study are available from the corresponding author on reasonable request.

## Ethics Statement

The studies involving human participants were reviewed and approved by Hospital Pablo Tobon Uribe. Written informed consent for participation was not required for this study in accordance with the national legislation and the institutional requirements.

## Author Contributions

ARR and CBM were involved in the study design, data recollection and study conduct. All authors interpreted the results, were involved with the writing of the manuscript and approved the final draft.

## Funding

The medical writing of this article was funded by Novo Nordisk. The funders had no role in study design, data collection and analysis, decision to publish, or preparation of the manuscript.

## Conflict of Interest

The authors declare that the research was conducted in the absence of any commercial or financial relationships that could be construed as a potential conflict of interest.

## Publisher’s Note

All claims expressed in this article are solely those of the authors and do not necessarily represent those of their affiliated organizations, or those of the publisher, the editors and the reviewers. Any product that may be evaluated in this article, or claim that may be made by its manufacturer, is not guaranteed or endorsed by the publisher.
